# Three-Dimensional Semantic Segmentation of Pituitary Adenomas Based on the Deep Learning Framework-nnU-Net: A Clinical Perspective

**DOI:** 10.3390/mi12121473

**Published:** 2021-11-29

**Authors:** Xujun Shu, Yijie Zhou, Fangye Li, Tao Zhou, Xianghui Meng, Fuyu Wang, Zhizhong Zhang, Jian Pu, Bainan Xu

**Affiliations:** 1 Medical School of Chinese PLA, Beijing 100853, China; shukelson@msn.com; 2 Department of Neurosurgery, The First Medical Centre of Chinese PLA General Hospital, Beijing 100853, China; leefangye@126.com (F.L.); ZhouTaoPLAGH@126.com (T.Z.); MengXHui301@126.com (X.M.); wangfytxdy@126.com (F.W.); ZhangZhiz301@126.com (Z.Z.); 3 School of Computer Science, Fudan University, Shanghai 200433, China; 20110240083@fudan.edu.cn; 4 Institute of Science and Technology for Brain Inspired Intelligence, Fudan University, Shanghai 200433, China

**Keywords:** pituitary adenomas, deep learning, medical image segmentation, magnetic resonance imaging

## Abstract

This study developed and evaluated nnU-Net models for three-dimensional semantic segmentation of pituitary adenomas (PAs) from contrast-enhanced T1 (T1ce) images, with aims to train a deep learning-based model cost-effectively and apply it to clinical practice. Methods: This study was conducted in two phases. In phase one, two models were trained with nnUNet using distinct PA datasets. Model 1 was trained with 208 PAs in total, and model 2 was trained with 109 primary nonfunctional pituitary adenomas (NFPA). In phase two, the performances of the two models were investigated according to the Dice similarity coefficient (DSC) in the leave-out test dataset. Results: Both models performed well (DSC > 0.8) for PAs with volumes > 1000 mm^3^, but unsatisfactorily (DSC < 0.5) for PAs < 1000 mm^3^. Conclusions: Both nnU-Net models showed good segmentation performance for PAs > 1000 mm^3^ (75% of the dataset) and limited performance for PAs < 1000 mm^3^ (25% of the dataset). Model 2 trained with fewer samples was more cost-effective. We propose to combine the use of model-based segmentation for PA > 1000 mm^3^ and manual segmentation for PA < 1000 mm^3^ in clinical practice at the current stage.

## 1. Introduction

Pituitary adenomas (PAs) arise from the pituitary gland; they comprise 10–15% of primary brain tumors and are the third most common type of intracranial tumor [1]. Most of these tumors remain small and do not cause substantial harm or symptoms. However, many progress to cause hormonal and neurological problems. Magnetic resonance imaging (MRI) is the most commonly used modality to diagnose PAs. The morphologies and sizes of PAs vary dramatically on magnetic resonance images. PAs are often classified into microadenoma, macroadenoma, and giant adenoma by size, functional and nonfunctional by hormonal complications, and primary or recurrent by treatment history. Segmentation of PAs on MRI is a routine clinical task for treatment decisions, surgical planning, and radiation therapy [2,3]. However, PA segmentation may be challenging. First, microadenomas are <1 cm in diameter, most are functional PAs diagnosed at an early stage because of hormonal changes, and some do not exhibit sufficient size for detection by MRI [4]. Second, previous surgery involving a recurrent PA changes the anatomy of the sellar region, making it more difficult to distinguish the boundary between tumor and normal tissues on MRI [5]. Third, cystic changes within PAs lead to irregular tumors and heterogeneity in the tumor texture on MRI. Finally, aggressive PAs invade surrounding structures, such as the cavernous sinus, the third ventricle, and the skull base; this invasion changes the anatomy of the sellar region. Manual slicer-by-slicer segmentation of PAs is reliable in a clinical setting; however, it is time-consuming and laborious, particularly for large and giant adenomas [6]. Therefore, an automatic method for PA segmentation is preferable.

The earliest and most commonly used image segmentation method is threshold segmentation, which divides an image into a target region and a background region by setting a characteristic threshold. Threshold-based segmentation is commonly used to segment three-dimensional (3D) brain tumors with various intensities [7]. However, the threshold-based method is inadequately efficient, because PAs are not enhanced on post-contrast images. Traditional algorithms, such as graph-based and balloon-inflation algorithms, have been introduced to segment PAs, but the results require post-editing [8]. Medical image processing software, such as 3D Slicer (https://www.slicer.org, accessed on 12 July 2021) and OsiriX (https://www.osirix-viewer.com, accessed on 12 July 2021), have been reported to segment tumors and offer a semi-automated method to segment PAs, but post-editing remains necessary when using these software [9,10,11].

Deep learning was one of the ten breakthrough technologies of 2013 [12]. Deep learning is powerful and outperforms traditional algorithms in many fields, including medical image segmentation [13]. Convolutional neural networks or fully convolutional networks have been widely used in medical image segmentation because of their high efficiency and time savings [14,15,16,17]. However, most convolutional neural network methods can only process two-dimensional images, and most medical data used in clinical practice are composed of 3D volumes.

Initially designed for microscopic cell segmentation, U-Net architecture has efficient and robust learning features for many medical image segmentation tasks [18]. nnUNet (“no new network”) is a U-Net-based deep learning framework that has enabled successful 3D semantic segmentation of various biomedical image datasets and has been considered the strongest U-Net baseline. Compared with other deep learning frameworks, nnUNet is a holistic, fast, and data-efficient segmentation method that can be applied out-of-the-box without requiring user intervention. nnUNet is ideal for users who do not have the expertise, time, data, or computing resources that are required to adapt deep learning solutions to medical image segmentation applications [19].

As far as we know, 3D semantic PA segmentation using deep learning approaches in T1ce images has never been reported. In this study, we developed and evaluated the nnU-Net models to explore a cost-effective way to apply deep learning-based models to PA segmentation in clinical practice.

## 2. Materials and Methods

### 2.1. Patient Information

A database of 243 consecutive PA patients who underwent transsphenoidal endoscopic surgery was used in this study. We collected clinical data in two stages from the PLA General Hospital under the permission of the PLA General Hospital Ethics Committee. In stage 1, medical records and pathology results of 208 patients were retrospectively reviewed from July 2020 to April 2021. This group included 106 male patients and 102 female patients aged 15 to 80 years (mean, 49.7 years); it was used for training and validating the first model. Among the 208 PA patients, 109 (52.4%) were primary nonfunctional pituitary adenoma patients. The primary nonfunctional pituitary adenoma subgroup included 72 male patients and 37 female patients aged 15 to 76 years (mean, 53.1 years); it was used for training and validating the second model. In stage two, 35 consecutive PA cases were reviewed in May 2020. This group included 17 male patients and 18 female patients aged 15 to 78 years (mean, 52.6 years); it was used to test the performances of the two models.

### 2.2. Magnetic Resonance Image Dataset

Preoperative magnetic resonance images were obtained with a 1.5-T magnetic resonance scanner (Siemens Espree, Erlangen, Germany). Sagittal and coronal T2-weighted images, as well as axial post-contrast T1-weighted (T1ce) images, with 1-mm thickness were acquired. The magnetic resonance images were collected in DICOM format; T1ce images of the cases were used as the data samples for deep learning. The parameters for T1ce were field-of-view = 130 mm, slicer thickness = 1 mm, matrix size = 512 × 512 × 176, flip angle = 15°, echo time = 3.02 ms, repetition time = 1650 ms, and voxel dimensions = 0.997 × 0.997 × 1 mm^3^.

### 2.3. Tumor Segmentation

T1ce images in DICOM format were converted into NIFTI images (nii format) using MRIConvert software (Version 2.1, University of Oregon, Eugene, OR, USA). The PAs were manually segmented in a slice-by-slice manner on T1ce images using ITK-Snap software (Version 3.8, University of Pennsylvania, Philadelphia, PA, USA). Sagittal and coronal T2 images were reviewed when it was difficult to detect the microadenomas on T1ce images. PA segmentation of the 243 cases was performed by a neurosurgeon specializing in PA surgery, who had 14 years of experience. Two senior neurosurgeons specializing in PA surgery, as well as one experienced radiologist, also rated the segmentation results. Finally, the maximum diameter and volume of each PA were measured in ITK Snap.

### 2.4. nnU-Net Framework

**Figure 1:**
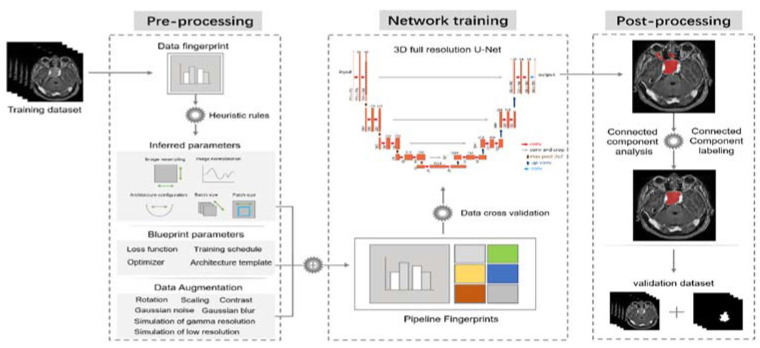
Flowchart of nnU-Net pipeline. To ingest training data, nnU-Net uses heuristic rules to determine the data-dependent hyper-parameters, referred to as the “data fingerprint”. Inferred parameters, blueprint parameters, and data fingerprints produce pipeline fingerprints, which produce network training for 3D U-Net using the hyper-parameters determined so far. The ensemble of network configurations, in combination with post-processing, determines the best average Dice coefficient for the training data.

We chose the nnU-Net as our segmentation network due to its ease of use and adaptability to diverse biomedical image datasets. nnU-Net is a deep learning-based segmentation method that automatically configures itself and executes the entire segmentation pipeline, including preprocessing, data augmentation, model training, and post-processing for any biomedical image dataset (Figure 1). The pipeline itself takes care of the hyper-parameter tuning and requires no change in the network architecture to achieve state-of-the-art results.

nnU-Net is free and open-sourced as an out-of-the-box segmentation tool. The source code is publicly available on Github (https://github.com/MIC-DKFZ/nnunet, accessed data: 12 July 2021). The software only requires a set of annotated magnetic resonance images as input data, as well as a mainstream computer with a powerful GPU. 

### 2.5. Study Design

**Figure 2:**
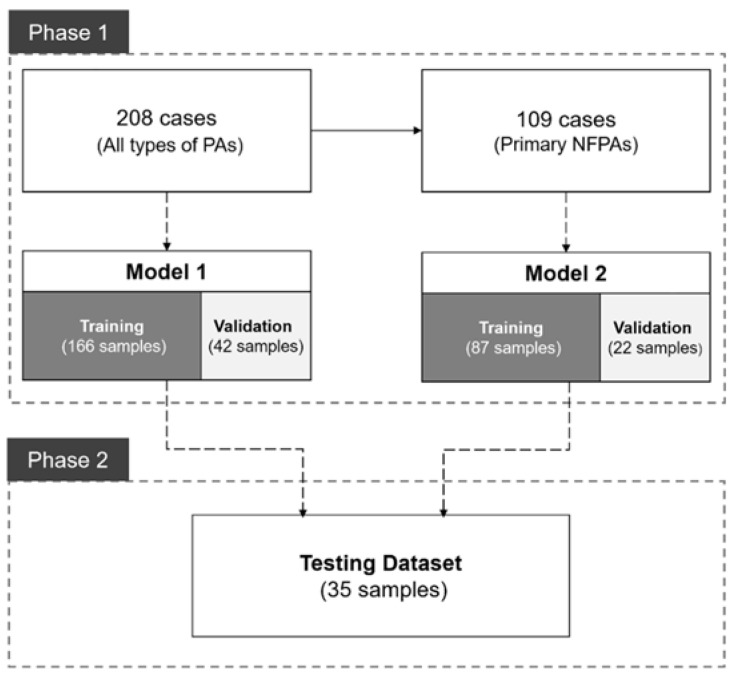
Schematic of the study design.

This study was conducted in two phases to utilize deep learning networks for 3D semantic segmentation of PAs, then to evaluate the performances of the models trained with different datasets (Figure 2). In phase one, two models were trained with the different datasets. The first model (Model 1) was trained and evaluated with all 208 PAs [80% (166 cases) training cases and 20% (42 cases) validation cases]. In total, 109 primary nonfunctional pituitary adenomas were used for the training and evaluation of the second model (Model 2) [80% (87 cases) training cases and 20% (22 cases) validation cases]. Cases in the validation dataset were not used for training in either model. In phase two, magnetic resonance images of 35 PA cases were collected consecutively in May 2021 as the testing dataset. The images were segmented and rated by the same clinicians who rated the first set of images. The performances of both models on the testing dataset were compared, and factors affecting model performance were examined.

## 3. Results

### 3.1. Patient Information and PA Characteristics

A database of 208 patients with PAs was included in the phase 1 of this study (Table 1): 106 (51%) patients were male and 102 (49%) were female; 168 (80.8%) were primary PA patients and 40 (19.2%) were recurrent PA patients. In total, 64.9% (135 cases) of the PAs were nonfunctional, and 35.1% (73 cases) were functional. Approximately 10.1% (21 cases) of the PAs were giant PAs, 78.8% (164 cases) were macroadenomas, and 11.1% (23 cases) were microadenomas. We divided the PAs into three groups: large (≥10,000 mm^3^), medium (1000–10,000 mm^3^), and small (≤1000 mm^3^). There were 11.1% (23 cases) in the large group, 25.5% (53 cases) in the small group, and 63.5% (132 cases) in the medium group.

### 3.2. Model Training and Evaluation

The nnU-Net models were realized with Python 3.8.5 and the Pytorch deep learning platform on a PC with an Intel Core i7-10700K CPU (3.8 GHz*16) and a GeForce RTX 3060 graphics card running the Linux OS (Ubuntu 16.04 STL).

**Figure 3:**
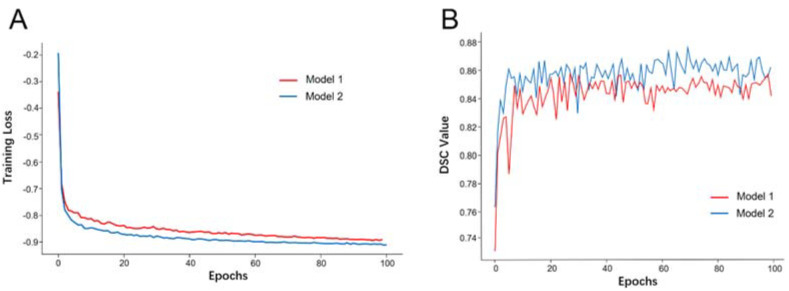
Loss curve of the training process for Models 1 and 2 (**A**). Evaluation metric curve of the training process for Models 1 and 2 (**B**).

The Dice similarity coefficient (DSC) was used to measure PA segmentation quality. The DSC quantifies the overlap between two PA subsets of manually segmented labels and model-prediction labels. As shown in Figure 3, Model 2 had better training loss and evaluation loss than did Model 1, according to the DSC metric. We stopped the training process at epoch 100 for both models, because we observed that further improvements in training loss and the DSC were insufficient for the extensive training time associated with greater numbers of epochs. Both models were trained for approximately 6 h.

### 3.3. Model Performance in the Validation Dataset

The mean DSC values of Models 1 and 2 were 0.803 and 0.853, respectively, for the validation dataset (Table 2). In the subgroup analysis, Model 2 offered a slight improvement in the DSC values over Model 1. Model 2 achieved improvements of 2% in the male group, 7% in the female group, 4% in the primary PA group, 2% in the nonfunctional pituitary adenoma group, 5% in the macroadenoma group, and 2% in the large volume group. In Model 1, the male group offered an improvement of 8% over the female group, while the improvement was 3% in Model 2. Both models offered DSC values > 0.8 for the medium and large-volume PA groups.

Model 1 only offered DSC values of 0.563 and 0.649 for the microadenoma and small PA groups, respectively. Model 2 was not evaluated in terms of microadenomas or small PAs because they were not included in the validation dataset.

### 3.4. Model Performance in the Testing Dataset

Thirty-five cases were collected consecutively as a testing dataset for both models; these cases were used to evaluate the difference in clinical performance between the two models. Table 3 presents the mean DSC values for the testing dataset and subgroup datasets of both models. Models 1 and 2 had DSC values of 0.7279 and 0.7284, respectively, for the testing dataset.

The DSC values of Models 1 and 2 for the testing dataset decreased by 8% and 10%, respectively, compared with the DSC values for the validation dataset. In the subgroup analysis, the DSC value of the male group was 12.6% higher than the DSC value of the female group in Model 1; it was 10.8% higher in Model 2. The DSC value of the primary PA group was 4.2% lower than the DSC value of the recurrent PA group in Model 1; it was 7.2% lower in Model 2. The DSC value of the nonfunctional PA group was 20% higher than the DSC value of the functional PA group in both models. Model 1 offered a DSC value of 0.843 in the giant PA group, 0.821 in the macroadenoma group, and 0.451 in the microadenoma group. Model 2 had a DSC value of 0.799 in the giant PA group, 0.83 in the macroadenoma group, and 0.448 in the microadenoma group. The medium and large PA groups both had DSC values > 0.8 for both models. The small PA group had mean DSC values < 0.5 for both models.

### 3.5. Performance Comparison between the Two Models

**Figure 4:**
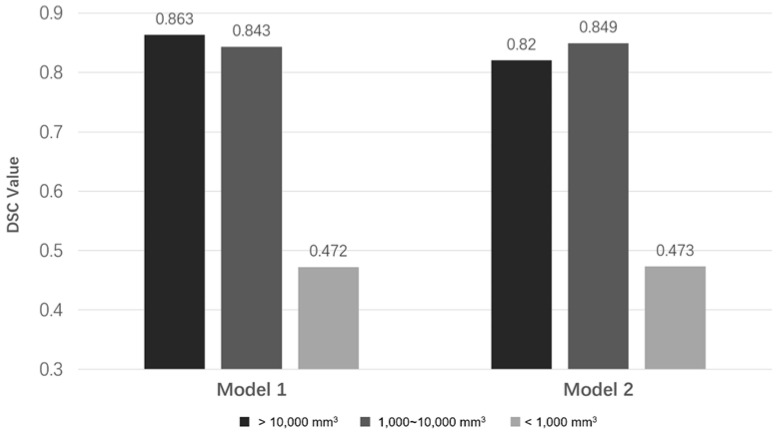
Performances of the models in different PA groups with different volumes in the testing dataset.

**Figure 5:**
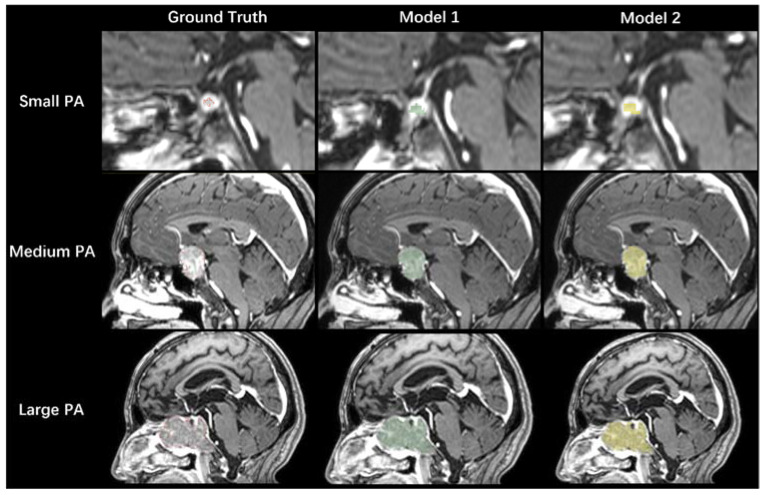
Three cases of small, medium, and large PAs are illustrated in rows. The first column represents the ground truth PA segmentations outlined in red, the second column represents PA segmentations from Model 1, and the third column represents PA segmentations from Model 2.

Model 2 was trained using the nonfunctional pituitary adenoma dataset, which was only half of the dataset used for Model 1. However, Model 2 offered a 5% improvement in the validation dataset DSC value, compared with Model 1; it provided the same DSC value (0.728) for the testing dataset (Table 2). According to the DSC values for the testing dataset, both models showed good performance for PAs > 1000 mm^3^, but they showed poor performance for small PAs (Figure 4). Figure 5 shows the segmentation results of Models 1 and 2 on PAs with different volumes.

### 3.6. The Relationship between DSC Values and PA Volumes

**Figure 6:**
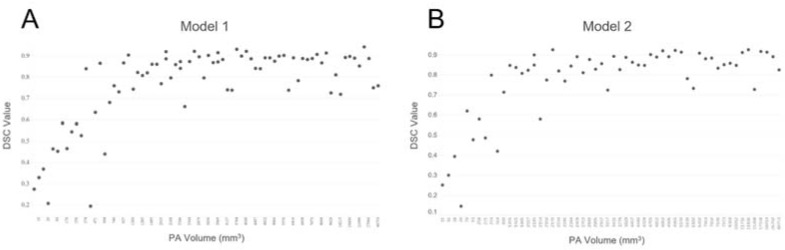
Distributions of DSC values and PA volumes in the validation and testing datasets for Model 1 (**A**) and Model 2 (**B**).

Figure 6 shows the relationship between the DSC values and PA volumes when the validation and testing datasets were considered together. The results indicated that the DSC values increased with PA volume in both models when the PA volume was <1000 mm^3^ and oscillated at >0.7 when the PA volume was >1000 mm^3^.

**Figure 7:**
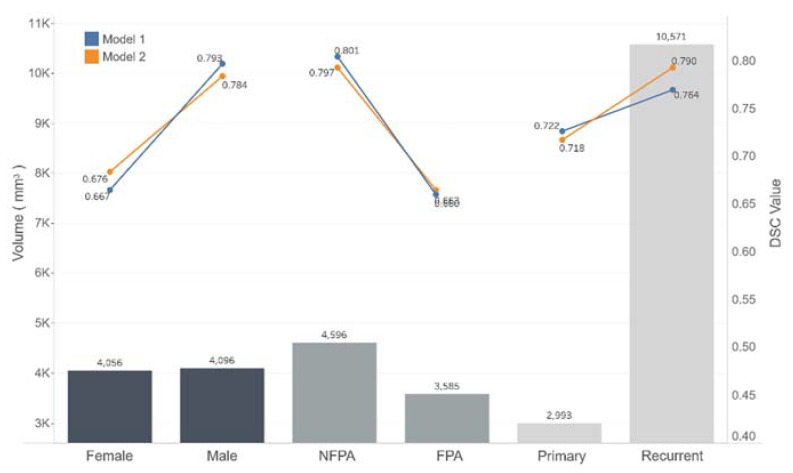
Relationships between mean volumes and DSC values of the male and female, NFPA and FPA, and primary and recurrent subgroups. DSC values increased with increasing mean volume in each subgroup. (NFPA: nonfunctional pituitary adenoma, FPA: functional pituitary adenoma).

Figure 7 shows the relationship between the DSC value and the mean volume of each subgroup; the DSC value tended to increase as the mean volume of each subgroup increased.

## 4. Discussion

PAs are some of the most frequently encountered benign intracranial tumors. Because of symptoms caused by hormones or the mass effect, PAs are often detected in various sizes and shapes on MRI. PA volume plays a crucial role in determining the initial treatment, tumor status, and subsequent management [6]. PA segmentation is a routine task for presurgical planning, intraoperative neuronavigation, radiotherapy, and post-treatment evaluation. Slice-by-slice manual segmentation of macroadenomas and giant adenomas is time-consuming; to our knowledge, automatic PA segmentation using deep learning has rarely been reported. Díaz-Pernas et al. used a multiscale convolutional neural network to classify and segment brain tumors. The DSC of PA segmentation in their study was 0.813 when using two-dimensional sagittal magnetic resonance images [15]. In our study, we applied the nnUNet deep learning framework to achieve 3D semantic segmentation of PAs with two models using different datasets. Models 1 and 2 offered DSC values of 0.803 and 0.853 for the validation dataset, respectively. In comparison with traditional algorithms, graph-based and balloon inflation methods were reported to have DSC values of 0.777 and 0.760, respectively [8].

Model performance depends on three factors. First, the configuration of hyper-parameters in the model must be optimized to achieve optimal performance. Second, big data is a boon for deep learning. The inclusion of more data leads to better performance. Third, the data distribution in the dataset affects model performance. In this study, because nnUNet automatically configured itself for both models, the data volume and distribution were related directly to model performance. Model 2 had a training dataset that was almost half the size of Model 1, but it provided a 5% increase in the DSC value during phase 1 (Table 2). This does not indicate that the performance of Model 2 was better than the performance of Model 1. Because there were no microadenomas in the validation dataset of Model 2, the data distribution was the leading cause of the difference in DSC values between the two models. When provided with the same testing dataset in phase 2, both models achieved the same DSC value of 0.728 and revealed nearly identical results in the PA subgroup analysis (Table 3). The DSC values for the testing dataset decreased by 7.5% for Model 1 and 12.5% for Model 2, compared with the DSC values for the validation dataset. The decrease in the DSC values was also related to the data distribution because 31.4% (11 of 35 cases) were small PAs in the testing dataset, while 23.8% (10 of 42 cases) for Model 1 and 0% (none) for Model 2 were small PAs in the validation dataset. In our study, the proportion of small PAs in the dataset affected the DSC values in the nnUNet models.

Tumor volume, which more accurately represents tumor size, was one of the most crucial factors that affected the performances of our nnUNet models. As Figure 6 shows, the DSC values increased with PA volume in both models when the PA volume was <1000 mm^3^ and oscillated at >0.7 when the PA volume was >1000 mm^3^. In the subgroup analysis (Figure 7), the DSC values differed between the male and female, primary and recurrent, and functional and non-functional groups. The mean volume difference in the subgroups suggests that functional adenomas are often detected during an early stage when they are small because of hormonal changes. The incidence of prolactin adenomas is higher in women than in men, and physicians tend to use a “wait and see” strategy with recurrent adenomas until they grow sufficiently large to cause new symptoms.

There are two reasons for the poor DSC values of small PAs. First, we chose the DSC value to evaluate model performance because it is the most widely used metric for validating 3D medical image segmentation. However, the DSC is sensitive to segment size, because it penalizes errors more in small segments than in large segments [20]. Some authors have proposed a new evaluation metric for segmentation performance, which emphasizes the small segments by assigning a higher weight to pixels in small lesions [21]. Second, some PAs are of insufficient size to be discerned by MRI, which causes inaccurate tumor labeling. Some authors have used a positron emission tomography-based adaptive threshold segmentation method for delineating small PAs to solve this problem [5].

Although deep learning has advanced in recent years, it remains difficult to train a deep learning model with big data that will perform well on all PAs and be applied universally in clinical practice. There are many challenges. First, big data collection requires open access to image databases from multiple hospitals, which is hampered by data privacy and security [12]. Second, datasets of PAs from clinical settings are biased, because small and giant PAs comprise a smaller proportion of cases than do medium-sized PAs; thus, an extended period is needed to collect sufficient numbers of small and giant PAs for model training. Third, the locations or boundaries of small PAs are difficult to discern on MRI; therefore, new imaging techniques are needed to solve this problem. Fourth, annotation and labeling of medical images for deep learning is labor-intensive, and the cost is incalculable. Based on these factors and considering the cost-effectiveness, training a deep learning model with a limited dataset that would perform well on most PAs for a single-center application is preferred.

In this study, Model 2 was trained with the nonfunctional pituitary adenoma dataset based on the following three considerations. First, nonfunctional pituitary adenomas are the most common PA subtype requiring transsphenoidal surgery. In this study, 109 (52.4%) of 208 PAs were nonfunctional pituitary adenomas. Second, nonfunctional pituitary adenomas generally appear regularly shaped, medium-sized, and have a homogenous texture on T1ce images; thus, they represent most PA features and can facilitate accurate tumor delineation as a ground truth label. Although it was trained with the half-sized dataset of Model 1, Model 2 had a generalization ability almost identical to the ability of Model 1 in the validation and testing datasets. As shown in Table 2 and Figure 4, Model 2 performed well for PA cases with volumes >1000 mm^3^, covering approximately 75% of the PA dataset. It is reasonable to combine the two methods in clinical applications, because Model 2 offered automatic and accurate segmentation results for medium and large PAs, while manual segmentation for small PAs was rapid and reliable. Furthermore, this pattern would generate more PA data with labels that can be used for model iterations, permitting continuous improvement of the model.

The limitation of this study is the size and scope of our PA dataset for deep learning. Model performance of nnU-Net might be improved with more small-size PA data. Another limitation is that our PA dataset is collected from a single medical center, which limits the performance of our model on data from other centers. Furthermore, other technologies, such as transfer learning, self-supervised learning, and federal learning might be good directions to make deep learning more promising in medical fields.

## 5. Conclusions

In this study, we developed and fully examined nnU-Net models for PA segmentation on the T1ce MRI image dataset. Models 1 and 2 were trained with different datasets, and both offered satisfactory segmentation results of PAs with volumes >1000 mm^3^. Model 2 trained with less samples was more cost-effective and practical in clinical practice, compared with Model 1. As the model performance of nnU-Net was related to the PA size, segmentation of small PA still remains a challenge. For PA segmentation in current clinical practice, we propose combining model-based and manual approaches.

## Figures and Tables

**Table 1:** Data information of patients and pituitary adenomas (PAs) in the Phase 1.

Patient Information and PA Characteristics	No. of Cases (%)
**Gender**	
Male	106 (51.0%)
Female	102 (49.0%)
**Primary/Recurrent PAs**	
Primary	168 (80.8%)
Recurrent	40 (19.2%)
**Nonfunctional PAs**	135 (64.9%)
**Functional PAs**	73 (35.1%)
ACTH	24 (11.5%)
GH	28 (13.5%)
PRL	16 (7.6%)
TSH	5 (2.4%)
**Size**	
Giant-PAs (≥4 cm)	21 (10.1%)
Macro-PAs (1 cm~4 cm);	164 (78.8%)
Micro-PAs (≤1 cm)	23 (11.1%)
**Volume**	
Large (≥10,000 mm^3^)	23 (11.1%)
Medium (10,000~1000 mm^3^)	132 (63.5%)
Small (≤1000 mm^3^)	53 (25.5%)
**Total**	208

**Table 2:** The mean Dice Similarity Coefficient (DSC) on validation dataset of both models.

	Model 1			Model 2		
	Train	Validation	Mean DSC	Train	Validation	Mean DSC
**Gender**						
Male	82	24	0.838	58	14	0.864
Female	84	18	0.756	29	8	0.833
**Primary/Recurrent PAs**						
Primary	136	32	0.808	87	22	0.853
Recurrent	30	10	0.787	-	-	-
**Nonfunctional PAs**	107	28	0.828	87	22	0.853
**Functional PAs**	59	14	0.753	-	-	-
ACTH	20	4	0.709	-	-	-
GH	22	6	0.729	-	-	-
PRL	14	2	0.768	-	-	-
TSH	3	2	0.896	-	-	-
**Size**						
Giant PAs	13	8	0.832	6	2	0.820
Macroadenomas	132	32	0.811	78	20	0.856
Microadenomas	21	2	0.563	3	0	-
**Volume**						
Large (≥10,000 mm^3^)	16	7	0.847	10	5	0.873
Medium (1000~10,000 mm^3^)	107	25	0.852	62	17	0.847
Small (≤1000 mm^3^)	43	10	0.649	15	0	-
**Total**	166	42	0.803	87	22	0.853

**Table 3:** Model performance of both models on the testing dataset.

	Testing Dataset	Model 1	Model 2
		Mean DSC	Mean DSC
**Gender**			
Male	17	0.793	0.784
Female	18	0.667	0.676
**Primary/Recurrent PAs**			
Primary	30	0.722	0.718
Recurrent	5	0.764	0.790
**Nonfunctional PAs**	17	0.801	0.797
**Functional PAs**	18	0.660	0.663
ACTH	10	0.679	0.672
GH	4	0.659	0.688
PRL	4	0.614	0.615
TSH	-	-	-
**Size**			
Giant PAs	4	0.843	0.799
Macroadenomas	22	0.821	0.830
Microadenomas	9	0.451	0.448
**Volume**			
Large (≥10,000 mm^3^)	3	0.863	0.820
Medium (1000~10,000 mm^3^)	21	0.843	0.849
Small (≤1000 mm^3^)	11	0.472	0.473
**Total**	35	0.7279	0.7284

## Data Availability

The data presented in this study are available on request from the corresponding author. The data are not publicly available due to the data also forms part of an ongoing study.
